# Interactions Between Anti-Angiogenic Therapy and Immunotherapy in Glioblastoma

**DOI:** 10.3389/fonc.2021.812916

**Published:** 2022-01-12

**Authors:** Saket Jain, Eric J. Chalif, Manish K. Aghi

**Affiliations:** Department of Neurological Surgery, University of California, San Francisco, San Francisco, CA, United States

**Keywords:** glioblastoma, glioma, anti-angiogenic therapy, bevacizumab, immunotherapy, checkpoint inhibitors, combinatorial therapy

## Abstract

Glioblastoma is the most aggressive brain tumor with a median survival ranging from 6.2 to 16.7 months. The complex interactions between the tumor and the cells of tumor microenvironment leads to tumor evolution which ultimately results in treatment failure. Immunotherapy has shown great potential in the treatment of solid tumors but has been less effective in treating glioblastoma. Failure of immunotherapy in glioblastoma has been attributed to low T-cell infiltration in glioblastoma and dysfunction of the T-cells that are present in the glioblastoma microenvironment. Recent advances in single-cell sequencing have increased our understanding of the transcriptional changes in the tumor microenvironment pre and post-treatment. Another treatment modality targeting the tumor microenvironment that has failed in glioblastoma has been anti-angiogenic therapy such as the VEGF neutralizing antibody bevacizumab, which did not improve survival in randomized clinical trials. Interestingly, the immunosuppressed microenvironment and abnormal vasculature of glioblastoma interact in ways that suggest the potential for synergy between these two therapeutic modalities that have failed individually. Abnormal tumor vasculature has been associated with immune evasion and the creation of an immunosuppressive microenvironment, suggesting that inhibiting pro-angiogenic factors like VEGF can increase infiltration of effector immune cells into the tumor microenvironment. Remodeling of the tumor vasculature by inhibiting VEGFR2 has also been shown to improve the efficacy of PDL1 cancer immunotherapy in mouse models of different cancers. In this review, we discuss the recent developments in our understanding of the glioblastoma tumor microenvironment specially the tumor vasculature and its interactions with the immune cells, and opportunities to target these interactions therapeutically. Combining anti-angiogenic and immunotherapy in glioblastoma has the potential to unlock these therapeutic modalities and impact the survival of patients with this devastating cancer.

## Introduction

Glioblastoma (GBM) is the most common primary brain malignancy in adults, comprising nearly 50% of all primary central nervous system (CNS) cancer, with an average annual incidence of 3.22 per 100,000 people (1). Despite decades of research that has improved our functional understanding of the molecular and genetic characteristics of GBM, there has been minimal improvement in overall survival, as evidenced by the dismal long-term survival ranging from 6.2 to 16.7 months in patients receiving trimodal therapy (2, 3). Unfortunately, new classes of medications that have revolutionized treatment for cancer outside of the CNS have so far been unsuccessful in clinical trials for GBM. Two classes of drugs that have failed phase III trials in GBM are checkpoint inhibitors and vascular endothelial growth factor (VEGF) inhibitors. These failures occurred despite the fact that targeting the immune system and angiogenesis were particularly promising candidates for the treatment of GBM due to its marked local immunosuppression and propensity for angiogenesis. Interestingly, recent studies have suggested that substantial interactions exist between immunotherapies and anti-angiogenic therapies in many cancers, including GBM. Understanding this interplay may lead to the development of improved and synergistic combinatorial therapies. In this review, we summarize the latest insights researchers have produce on the immunologic and angiogenic components of the GBM microenvironment with a particular emphasis on how immune and anti-angiogenic therapies might interact in GBM. We also review existing agents that are currently undergoing investigation as targeted unimodal or combinatorial therapy in GBM.

## The Immune Microenvironment of GBM

The microenvironment of the normal brain is generally immunosuppressive and was formally considered an immune privileged organ before the discovery of lymphatic vasculature lining murine dural sinuses (4). Despite this, the brain remains an immunologically unique organ as peripheral immune cells will only rarely patrol through the functional blood brain barrier (BBB). This BBB is composed of endothelial cells held together by intercellular tight junctions that restrict entry of most immune cells, and those cells that do cross will rapidly exit unless they have recognized a local antigen. As GBM grow beyond 1-2mm in diameter, the BBB becomes compromised allowing for a more robust infiltration of immune cells (5). Despite BBB breakdown and increased immune cell entry, GBM avoids targeting by immune cell through a number of mechanisms including local T-cell exhaustion, low tumor mutation burden, high heterogeneity among tumor cells, as well as release of a variety of soluble factors that lead to low levels of local and circulating immune cells.

CD8+ T cells in GBM are characteristically exhausted as a result of persistent stimulation. Exhaustion represents a unique transcriptomal profile that leads to an up-regulation of inhibitory immune checkpoints that ultimately leads to cell senescence (6). These dysfunctional T cells are classically identified by downregulated CD27/28 and upregulated CD57 and immune checkpoint receptors, which are accompanied molecularly by a decrease in proliferation and cellular metabolism, impaired response to cytokines, and eventual apoptotic death (7–10). One mechanism by which tumor cells provoke T cell death is through expression of Fas-L, which binds to Fas death receptor on T cells and leads to a caspase-mediated apoptotic pathway (11). GBM tumor cells often also express such checkpoint proteins as programmed death ligand 1 (PD-L1) and CTLA-4 for which increased levels of expression predict a worse clinical prognosis (12). Various transcription factors have been implicated in T cell exhaustion such as PBX3, Prdm1, Eomes family, CD122, and others that collectively contribute to changes in phenotype towards loss of effector function (6).

Tumor mutational burden (TMB) is defined as the total number of protein-altering mutations in coding regions of genes. In many cancers, a high TMB is associated with an immune-reactive phenotype and substantial local cytotoxic CD8+ cell population (13). GBM has a lower TMB phenotype than most other solid malignancies, which likely contributes to its poor prognosis due to fewer immunogenic neoantigens to provoke immune response. Unexpectedly, a higher relative TMB among patients with GBM confers a worse prognosis on survival and worse response to immunotherapy, which opposes the trend seen in most other cancers (14–16).

GBM also has profound intratumoral heterogeneity as characterized by intercellular genetic diversity within the tumor. Previously canonical subtypes of GBM (proneural, neural, mesenchymal, classical) have recently been challenged as evidence from single cell sequencing data reveals that these subtypes are all variably expressed within the same GBM sample, which reflects the heterogeneity of different spatial compartments in the tumor with molecular classifications that likely exist on a continuum rather than binary form (17, 18). High intratumoral heterogeneity results in inconsistent molecular targets whereby divergent tumor cells will not respond similarly to certain therapies.

GBM tumor cells release various cytokines that contribute to the immunosuppressive milieu including IL-1/IL-6/IL-10 (suppresses activity of CD8+ and Th cells) (19–21), chemokine CCL22 (attracts CD25+ FoxP3+ regulatory T cells to the tumor niche) (22, 23), and TGF-β (facilitates epithelial−to−mesenchymal transition and impedes transmigration of T cells to the tumor *via* the downregulation of ICAM expression on the endothelial cell surface) (24–26). In addition to local immunosuppression, there is systemic immune impairment as indicated by decreased levels of circulating T cells and increased proportion of regulatory T cells measured in the peripheral blood (27). Systemic immunosuppression as measured by high neutrophil to lymphocyte ratio (NLR) is a negative prognostic factor on overall survival and progression-free survival (28).

## The Vascular Microenvironment of GBM

Extensive angiogenesis is characteristic of glioblastoma and is controlled by a number of converging pathways. Glial stem cells are one of the main driver of angiogenesis. They serve vital functions in providing blood supply and are identified by the fraction of GBM expressing CD133+. One mechanism by which glial stem cells route blood to tumor is through upregulation of genes involved in angiogenesis such as release of vascular endothelial growth factor (VEGF) (29). Endothelial cells, in turn, promote adjacent phenotypic change towards tumor stems cell *via* NOTCH ligand expression as well as release of nitric oxide to activate notch signaling (30, 31). This results in a positive feedback loop between stem cells and the blood vessel wall, promoting rapid angiogenesis. Additionally, tumor stem cells may miraculously themselves differentiate to endothelium, functionally assisting in the formation of competent microvessels (32, 33). Interestingly, tumor-derived endothelial cells are more prevalent within the core of the glioblastoma than the tumor periphery. This likely speaks to adaptation responses allowing these cells to survive in more stressful conditions than normally derived vasculature. Pericytes have also been reported to derive from the same cell lineage as GBM stem cells (34). GBM has intensive metabolic demands, and there is often local tissue hypoxia due to insufficient oxygen supply. Hypoxia drives expression of tumor stem cells genes such as those involved in the Notch pathway and calcineurin pathway (35). Hypoxia-inducible factor 2 alpha (HIF-2a) is the driver of stem cell change in response to hypoxia, and unlike HIF-a, which promotes apoptosis, HIF-2a promotes resilience in low oxygen conditions (36). HIF-2a also leads to upregulated transcription of VEG-F.

These studies collectively provide intriguing evidence that tumor blood vessels themselves are neoplastic and capable of actively remodeling the perivascular niche.

Vessel co-option is another means by which GBM cells can gain access to oxygen and nutrients. In this process, GBM cells grow towards and then along existing vasculature within the brain. In particular, GBM grow in areas where there is large surface area for tumor to endothelial cell contact, such as between micro vessels that run parallel to each other, among capillary loops, or near dilated capillaries (37). One essential driver of vessel co-option is WNT-7 expression, a pathway promoted within Oligodendrocyte-precursor stem cells (38). An important chemokine for co-option is bradykinin, which is released by endothelial cells and serves as a chemoattract to tumor cells (39).

## Current Immunotherapies for GBM

A variety of immunotherapies for have been tested in phase I, II, and III clinical trials. These therapies generally fit into the following categories: targeted molecular inhibitors, vaccine-based therapies, viral therapies, and adoptive T-cell therapies. While no individual or combination of immunotherapy for GBM has so far been successful in phase III testing, a number show promise in certain subgroups of patients and these are currently being further investigated. One difficulty in testing new therapies is the relative few number of patients that present with GBM in comparison to the total number of therapies on trial. To remedy this, many trials have begun to use historical control groups and may combine clinical phase I and II or phase II and III testing in certain cases (40–43).

### Targeted Molecular Inhibitors

Immune checkpoint inhibitors (CPI) are the most well studied molecular inhibitors in GBM and they have shown impressive increases in survival for a number of other cancer types (44). These drugs target inhibitory receptors expressed by immune cells or their ligands. The most well-studied CPIs target programmed cell death protein 1 (PD-1), programmed death-ligand 1 (PD-L1) and cytotoxic T-lymphocyte-associated protein 4 (CTLA-4). T-cell immunoglobulin and mucin domain 3 (Tim-3) and Lymphocyte-activation gene 3 (LAG-3) are more recently investigated CPIs that have shown to be co-expressed with classic CPIs and show promise as additional targets in clinical testing (45–50).

The failure so far for CPI to promote survival in GBM, despite its efficacy in other cancer types, is likely a combination of the many unique facets of the GBM immune landscape as described above (51). Briefly, it is a heterogenous tumor with low tumor mutational burden and persistently exhausted tumor-associated lymphocytes. As such, an incomplete immune response is mounted with subsequent selection of tumor cells best able to respond to CPI. There also exists an intricate balance between pro and antitumoral immune regulation, whereby targeting one immune checkpoint receptor results in immediate recalibration of other signaling pathways to re-center the balance and prevent immune overactivity. To overcome varied methods of resistance, CPI in combination with other targeted molecular inhibitors holds future promise as these therapies may work synergistically to target select pathways that GBM utilizes to overcome CPI monotherapy.

A tryptophan metabolic enzyme, IDO is also considered a contributing factor for immune resistance in GBM through tryptophan metabolism. A recent study has shown that IDO can have shown that IDO can suppress immune response by inducing the expression of compliment factor H (CFH) independent of tryptophan metabolism and could act as a potential target for therapy (52).

Interactions of the tumor cells with the cells of the tumor microenvironment has led to the discovery of novel targets for therapies. A recent preclinical study showed that targeting tumor associated macrophages (TAMs) using a colony stimulating factor 1 receptor (CSF-1R) combined with radiation results in increased survival of mice (53). A recent study from our lab identified cancer associated fibroblasts (CAFs) in GBM and showed that pro-tumoral effects of CAFs are mediated through osteopontin and HGF pathway in GBM (54). STAT3, a member of STAT family of transcription factors has been shown to have an important role in regulating the GBM tumor microenvironment and is considered a promising target (55).

### Vaccine-Based Therapies

Peptide, dendritic cell, and heat shock protein vaccines are the primary vaccine types in GBM treatment. Peptide vaccines consist of the direct inoculation of tumor associated antigens (TAA). These peptides can be extracted from patient tumor tissue, or from synthetic production of canonical GBM epitopes. Commonly targeted GBM TAAs are epidermal growth factor receptor variant III (EGFRvIII), gp100, survivin, TRP-2, AIM-2, MAGE-1 (56). One of the difficulties of peptide vaccines is that individual use is restricted to certain HLA haplotypes, which limits generalizability of these agents, and creates a substantial hurdle to their testing in clinical trials (57).

Among the challenges of vaccine therapy are the heterogeneity of tumor cell populations that may not all hold the same mutations. As such, there will be a selection towards those cells that do not express selected TAA. Furthermore, there is a large population of MHC I−absent GBM tumor cells that will not respond to vaccine approaches because they do not present antigens. Lastly, the local GBM landscape is T cell depleted, so developing therapies to enhance T cell infiltration to tumor will be necessary in combinatorial approaches to augment the efficacy of vaccine approaches. In general, because of their minimal toxicity, there is little risk in adding vaccines on top of other chemotherapeutic or targeted modalities.

### Viral Therapies

Viral based therapies tackle the immunosuppressive GBM microenvironment through direct oncolysis and delivery of therapeutic payload or gene therapy to the tumor. These are generally delivered intratumorally or postoperatively into the resection cavity, and specifically target GBM due to its high metabolic activity and rapid cell cycle progression in comparison to surrounding brain parenchyma. This approach has the unique benefit that the viral vector itself will often stimulate an immune response that an immunostimulatory transgene within the virus can further potentiate. Subsequent tumor cell death in presence of activated immune cells will theoretically allow tumor cells antigens to be processed and subsequently targeted by immune cells. A variety of virus types have reached clinical trials including retrovirus, adenovirus, lentivirus, herpes simplex virus, and reovirus, parvovirus, measles virus, poliovirus, and others (58, 59). The few viral therapies that have reached phase 3 trials have failed to demonstrate positive effect on overall survival (60–63). Additionally, the most thoroughly investigated therapies delivered the suicide genes thymidine kinase or cytosine deaminase (toca 511) rather than agents that may more directly modulate the local immune landscape. There are promising agents on the horizon in preclinical, phase 1, and phase 2 studies that directly deliver immunomodulatory agents such as ad-RTS-hil-12, interferon beta, VB111 (discussed below), tesurpaterev (64), RLI, and others (59, 62, 65).

### Adoptive T-Cell Therapies

Adoptive T cell therapy (ATC) is a setup by which autologous T cells are extracted from patient, expanded *in vitro*, and subsequently returned to patient in larger numbers. More recently, there have been efforts to genetically modify extracted T cells to express specific antigen or tumor receptors. Clinical trials for ATC lag behind other approaches and none have yet reached phase III testing.

## Current Anti-Angiogenic Therapies for GBM

Most anti-angiogenic therapies target ligands, their receptors, or downstream signaling pathways that are implicated in vessel growth. The primary driver of angiogenesis in GBM is VEGF-A, which is secreted by tumor cells and binds to receptor VEGFR-2 on the endothelial cell surface, resulting in the activation of PI3K–Akt and MAP kinase pathways that promote endothelial cell proliferation and survival. Weaker proangiogenic growth factors are platelet derived growth factor (PDGF) that binds to PDGFRa/b and fetal growth factor (FGF) that binds to FGFR, as well as cell surface targets such as Notch and αvβ3/αvβ5 integrins. Targeting the above proteins or their implicated intracellular signaling proteins has been an active area of investigation.

### Antibody Therapies

Bevacizumab, a monoclonal antibody that targets free VEGF-A, is the only targeted therapy that has received FDA approval for GBM. It was originally granted accelerated approval in 2009 for recurrent GBM based on the success in prolonging patient survival in two phase II clinical trials (66, 67). Thereafter, bevacizumab had disappointing results for newly diagnosed GBM in randomized phase III clinical trials AVAglio and RTOG 0825, in which there was no improvement in overall survival (68, 69). Despite this, FDA converted bevacizumab to full approval for recurrent GBM due to a reduction in disease progression based on findings from another phase III study, EORTC 26101 (70). Bevacizumab’s failure to improve OS despite its prolongation of progression free survival is likely due to impressive improvements in imaging that are merely artifact changes in blood flow (via rapid reduction in vessel permeability and contrast extravasation) rather than true treatment effect on tumor biology. However, bevacizumab has been shown to result in reduction in the use of corticosteroids to treat brain edema. There are many other antibody therapies that are being investigated, including those targeting other growth factors (HGH, PDGF, PGF, etc), their receptors (VEGFR-2, EGFR, PDGFR, etc), as well as decoy receptors (VEGF-trap), but none so far have had successful phase three trials.

### Resistant Mechanisms to Anti-Angiogenic Therapies

Mechanisms of resistance to antibody therapy are manifold and include converging adaptive and intrinsic mechanisms centered on upregulation of alternative or redundant angiogenic pathways, protection of tumor vasculature by recruiting proangiogenic cells such as pericytes, increased invasiveness of tumor cells that further co-opt normal brain vasculature, increased metastatic seeding, selection and propagation of those tumor subpopulations that avoid inhibition, and myeloid cells that release alternative proangiogenic factors (71, 72). Furthermore, one study found that about 20% of primary GBM do not express VEGFA and as such would likely not at all respond to anti-VEGF treatment (73). Microarray and single-cell sequencing of bevacizumab-resistant patient glioblastoma specimens demonstrates upregulated mesenchymal genes, particularly β1 integrin glycoprotein, receptor tyrosine kinase c-Met, YKL-40, and transcription factor ZEB1 (74–76). Glucose transporter 3 (GLUT3) also appears to play a vital role in antiangiogenic therapy resistance, and inhibiting this protein resulted in cell death in bevacizumab-resistant GBM cells (77). To combat rapid resistance, a number of other targets have been developed including tyrosine kinase inhibitors, signal pathway inhibitors, and novel targeted therapies that can be used singly or in combination to target vasculature in a multifaceted approach. These have begun to be employed in combination with bevacizumab to target tumor invasion and angiogenesis (78–81).

### Tyrosine Kinase Inhibitors

Small molecule inhibitors are an alternate way to target growth factor ligands and their receptors. Unlike the fine selection that antibody therapy has on its target, small molecule inhibitors of tyrosine kinase will variably target several tyrosine kinase receptors that together impact vessel growth. For instance, the best studied tyrosine kinase inhibitor cediranib targets VEGFR-1/2/3, PDGFR- α/β, FGFR-1, EGFR, as well as the stem cell factor c-kit receptor (82–84). However, cediranib has failed phase III clinical testing in prolonging progression free survival in patients with recurrent GBM (84). One of the particular difficulties of small molecule inhibitors in the treatment of GBM is the relatively impermeable BBB that heavily restricts delivery of these molecules to the tumor. It has been demonstrated that many tyrosine kinase inhibitors are indeed substrates of P-glycoproteins and other resistance proteins that are highly expressed on capillary endothelial cells and are involved in active efflux of drugs out of the CNS. Additionally, while small molecule inhibitors often inhibit multiple types of tyrosine kinase, in general they are insufficient to block all receptor signaling, and as a result GBM may simply respond by upregulating or activating these same tyrosine kinase receptors (85).

### Miscellaneous Agents

A diverse set of other agents have been developed to target vascular growth *via* unique mechanisms. Some targets for inhibition include signaling pathways that are downstream of tyrosine kinase such as protein kinase C, mTOR, Ras, and others, which have proven successful in multiple other cancer types. For instance, thalidomide is being tested in glioma and it has been shown to inhibit EGF-induced phosphorylation of extracellular signal regulated kinase (ERK), as well as EGF-induced Ras activation by preventing transition to GTP-bound active Ras (86). There are intriguing other agents with mechanisms that function outside of the tyrosine kinase signaling pathway framework. These include cytokines and other soluble factors, extracellular ligands, as well as intracellular cell machinery with diverse and sometimes converging pathways. One such agent is celecoxib, a selective cyclooxygenase-2 (COX-2) inhibitor, which has been shown to reduce vascularization and subsequently suppress expression of proteins VEGF and HIF-1α (87). In a phase 2 study, however, the combination of thalidomide and celecoxib in addition to standard of care failed to meet the primary endpoint in reducing progression free survival, and also failed to correlate treatment response with a reduction in angiogenic peptides including VEGF (88). There are also hormonal therapies such as 2-methoxyestradiol, an estradiol metabolite, which downregulates HIF-1a at the posttranscriptional level and results in decreased HIF-1α-mediated VEGF expression. Results of a phase 2 trial showed modest anti-tumor effect (89).Another HIF factor HIF-2α has been studied in GBM and is associated with poor patient outcome (90). Recently, FDA approved a HIF-2α inhibitor belzutifan for hemangioblastomas a different central nervous system tumor.

Matrix metalloproteinases are found in tumor cells and are implicated in cell invasion by means of proteolytic degradation of extracellular proteins. There is evidence that these metalloproteinases facilitate the specific invasion associated with vessel cooption (91). In addition, matrix metalloproteinases are able to activate various cytokines, such as TGF-B and VEGF through direct interaction (92). Inhibitors of these metalloproteinases hold promise as anti-angiogenic agents in a variety of cancers, however one such agent marimastat failed phase 2 testing in newly diagnosed GBM patients (93).

Another promising agent is enzastaurin, an inhibitor of protein kinase Cβ (PKC-β). Anti-angiogenic effect of this drug is based around an alternate downstream VEGF signaling pathway that is essential for endothelial proliferation and migration. Inhibition of PKC-β by enzastaurin has been demonstrated to decrease microvascular density and VEGF expression in human tumor xenografts (94). The drug also causes direct cytotoxicity to tumor cells. After results in a phase 2 trial in which a germline polymorphism on chromosome 8 (DGM1) was found *post hoc* to be associated with a significant increase in response to enzastaurin in newly diagnosed GBM patients, it has been granted fast track approval for phase 3 testing in biomarker positive patients (95, 96).

Integrins are yet another molecular vascular target. These are highly expressed on the endothelial surface and interact with extracellular matrix proteins to promote endothelial cell migration. They also interact and with immunoglobulin superfamily molecules to promote pro-angiogenic macrophage trafficking to tumors (97). However, in phase 3 clinical testing, the addition of cilengitide—a cyclic RGD pentapeptide that selectively inhibits the integrins αvβ3, αvβ5 and α5β1—to temozolomide did not improve outcomes (98). Additionally, combination trials of cilengitide with cediranib, a VEGFR inhibitor, failed to produce good results (99).

## Synergy in Immunotherapy and Antiangiogenic Agents in GBM

Despite decades of developing new antiangiogenic agents and immunotherapies, none so far have successfully prolonged overall survival for newly diagnosed or recurrent GBM. However, some show promising results in certain subgroups (e.g. enzastaurin in GBM patients with the DGM1 polymorphism). While these results have been disappointing, there is optimism that combination therapies between agents that target the immune and vascular systems could be more successful. It has been demonstrated that there exists substantial crosstalk between the vascular and immune systems. Understanding how these interactions may potentiate drug effects will likely lead to the development of successful therapies for GBM in the future.

### Soluble Factors With Dual Immunologic and Angiogenic Functions

A variety of soluble factors have been demonstrated to influence both the immunologic and angiogenic aspects of the tumor microenvironment. One of these is VEGF, the primary driver of angiogenesis, which is also a potent immunosuppressive factor that promotes tumor growth by modulating the adaptive and innate immune compartments. VEGF affects the ability of CD34+ hemopoietic progenitor cells to differentiate into functional dendritic cells (DC) in an NF-kB signaling-dependent manner, thus contributing to evasion of immune survelience (100, 101). Those DCs that do develop in setting of VEGF have dramatically reduced functional capacity in presenting antigen to allogenic T cells or in stimulating a primary immune response with a presented antigen. Interestingly, VEGF does not affect function of already-mature DCs (102). These findings are corroborated in a report on GBM where VEGF blockade likewise led to more differentiated and less active DCs in the brain (103). VEGF enhances a number of inhibitory checkpoints involved in T cell exhaustion including PD-1, as Tim-3, CTLA-4, and Lag-3 (104). Data from colorectal cancer reveals that VEGF induces the expression of transcription factor TOX in T cells to drive an exhaustion-specific transcription program (105). VEGF also suppresses immune cell trafficking through the downregulation of various cell adhesion molecules including ICAM-1 and VCAM-1 (106, 107). VEGF has been demonstrated to promote the recruitment and proliferation of several immunosuppressive cells, including regulatory T cells and M2-like pro-tumoral macrophages (108, 109). VEGF may also effect systemic immune system, as demonstrated in mice subjected to VEGF infusion have decreased overall quantity of systemic DCs, T-cells, and B-cells as measured in spleen and lymph nodes (102). Other growth factors are also implicated dually in the immune and vascular compartments, such as FGF, which in addition to its potent anti-angiogenic properties, also attracts immunosuppressive immune cells such as myeloid-derived suppressor cells (MDSCs) as demonstrated in breast cancer (110). FGF also promotes M2 polarization (110).

TGF-β is another multifunctional cytokine that is implicated in immune and vascular escape mechanisms in GBM (111). TGF-β signaling stimulates production of VEGF and a number of other pro-angiogenic factors including HIF-1, FGF (112). TGF-β is in a signaling loop with proangiogenic metalloproteinases released by cancer cells, that lead to mutual upregulation and facilitates tumor progression, vessel cooptation, and proangiogenic state (113). Interestingly, TGF-β is also implicated in anti-angiogenic pathways, and it appears that competing mechanisms result in a fine balance in angiogenic signaling which is finely dependent on cell content (114). GBM and other malignancies predominantly exploit the pro-angiogenic signaling pathway (115).

TGF-β exerts strong immunosuppressive pro-tumoral effects on all cells in the immune system. TGF-β1 in particular has been demonstrated to potently block differentiation of immune cells to cytotoxic CD8^+^ cells or CD4^+^ cells, and also inhibits their function by suppressing the release of killing enzymes such as granzyme and perforin from CD8^+^ cells (111). It also directly inhibits MHC class I expression on glioma cells. Because of its broad implications in many pro-tumoral mechanisms, there are a number of inhibitors of TGF-β that are being tested as therapies for GBM (111).

### Immune Cells Influencing the Tumor Vasculature

Immune cells may regulate tumor angiogenesis by releasing soluble factors that generally promote vascular genesis. M2 macrophages produce a number of proangiogenic factors including growth factors (VEGF, EGF, FGF, PDGF, TGF-b), CXC/CCL chemokines, and ANGPT2 (116, 117). Likewise, CD8^+^ T-cells have been shown to upregulate a number of chemokines including CXCL9, CXCL10, and CXCL11, which collectively enhancing pericyte recruitment into the tumor microenvironment (118). In ovarian cancer, tumor-associated plasmacytoid dendritic cells induce angiogenesis *in vivo* through production of TNF-alpha and IL-8 (119). MDSCs and neutrophils may promote angiogenesis by producing matrix metalloproteinase 9 as well as Bv8, of which both have been demonstrated to promote release of VEGF (116, 120). Bv8 inhibition resulted in reduced tumor vasculature in several solid malignancies (121). MDSCs can also integrate into the vasculature itself, helping to create a stable and proliferative vessel wall (122). Regulatory T cells have been implicated as pro-angiogenic forces, and their depletion in ovarian cancer resulted in robust reduction of the VEGF as measured in tumor microenvironment (123). A number of other immune cells have been reported to release VEGF including several types of natural killer cells and B cells (117).

One intriguing report from patients with recurrent GBM shows that an increase in infiltrating tumor-associated macrophages after bevacizumab is associated with poor survival, which suggests that entry of these macrophages from peripheral blood to tumor may represent an escape mechanism from antiangiogenic therapy (124). Another report reveals that a relative downregulation of macrophage migration inhibitory factor exists in bevacizumab-resistant GBM xenografts compared to bevacizumab-naïve xenografts (125). The apparent difference in these findings likely speaks to the complex interplay between M1 and M2 differentiated phenotypes that are implicated in mechanisms of bevacizumab resistance, likely by a downregulation in total migrating macrophages, but relative proliferative expansion of M2 macrophages to promote tumor growth (125).

### Effects of the Abnormal Tumor Vasculature on Immune Cells

The dysfunctional vasculature present in most cancers generally prevents the activation of immune cells. Indeed, a “tumor-endothelial barrier” has been described by which tumor endothelial cells suppress T cells, target them for destruction, and block them from entering the tumor (126). As part of this barrier, tumor endothelial cells will downregulate a variety of integrins and other adhesion molecules necessary for immune cell margination and subsequent extravasation (107). Specifically, endothelin 1 was found to be upregulated in numerous immunosuppressed tumors, and mechanistically blocks T cell adhesion to the endothelium through production of nitric oxide resulting in the suppression of ICAM1 (127). The immunosuppressive mediator IDO expressed in endothelial cells can cause dilation of vessels mediated *via* nitric oxide in CNS tumors. While tumor vasculature suppresses entry of most immune cells, it has been demonstrated that immunosuppressive cells such as regulatory T cells are better able to migrate through endothelium, though mechanisms by which tumor selectively allows entry are still being investigated (128).

Those pro-inflammatory immune cells that manage to attach to endothelium are immunologically suppressed by a number of ligands on the endothelial surface, including inhibitory checkpoints and a reduction in MHC class I-presenting complexes. Specifically, endothelial cells have been found to express PD-L1 and PD-L2 that retain their function in downregulating CD8+ T cell activation and cytoxicity (129). In GBM, PD-L1 levels positively correlate with VEGF (130). Expression of TIM-3 has also been described to be upregulated in a number of cancer associated endothelium, including lymphoma, where it functioned to inhibit activation of CD4+ T cells and Th1 phenoytype polarization (131). Fas ligand has been demonstrated to be functionally competent on tumor endothelium. In one intriguing study, inhibition of VEGF resulted in tumor growth suppression by CD8+ T cells in manner that was dependent on the attenuation of FasL (132). The tumor endothelium also produces a number of anti-inflammatory cytokines including endothelin-1, FGF, TGF-beta, IL-6, IL-8, PDGF, G-CSF, and others (133).

### Individual Therapies That Target Both the Immune and Vascular Compartments of GBM

As is evident from the substantial crosstalk that exists between the immune environment and vasculature, any one targeted therapy will likely be implicated in a variety of mechanisms that have unintended effects on cancer biology. For instance, by blocking VEGF-A, bevacizumab may inhibit VEGF-mediated immune suppression by suppressing regulatory T cells or expression of immune checkpoints. Likewise, immune checkpoint inhibitors may suppress M2 phenotypic change, resulting in a decreased M2-mediated angiogenesis. In a similar manner, some therapeutic agents for GBM have been specifically designed as dual agents to target angiogenesis and activate the immune system. Chief among these is ofranergene obadenovec (VB-111), a replication-deficient adenovirus vector that carries a transgene for a chimeric death receptor composed of TNFα receptor connected to intracellular Fas (62). When TNFα binds to the chimeric receptor, Fas pathway leads to cell quiescence and death. This transgene is restricted however to angiogenic endothelial cells which nearly exclusively have an activated pre-proendothelin 1 (PPE-1)–3x promoter (63). Initiation of this therapy has shown to result in dramatic infiltration of CD8+ T cells in tumor tissue with subsequent cell apoptosis, which likely results dually from pathways downstream of chimeric death receptor in addition to immunogenic viral epitopes that stimulate immune targeting (134). While a phase III trial failed to demonstrate survival benefit of VB-111, the patients enrolled here did not receive a ‘priming’ dose of VB-111 that may prove necessary in synergistic success with bevacizumab, as demonstrated with the good results in prior phase II study that used such a ‘priming’ dose in the study design (135). Another treatment with overlapping effects are Ang-2 inhibitors, which have been developed after GBM treated with bevacizumab were shown to express higher Ang-2 levels (136). Intriguing results from preclinical glioma studies demonstrate the reprogramming of tumor associated macrophages from M2 to M1 phenotype that co-occurs with vessel density reduction during treatment with an Ang-2 inhibitor (136–138).

However, other drugs are being developed that accommodate obviously competing immunologic and vascular pathways in the tumor microenvironment. One such example is ABT-510, a thrombospondin-1 (TSP-1) mimetic drug that competes with TSP-1 and inhibits glioma angiogenesis *in vivo* (139). The receptor for TSP-1, CD36, is upregulated in antigen presenting cells such as tumor associated macrophages and dendritic cells (140). Targeting this receptor for inhibition may therefore inadvertently augment the immunosuppressive local milieu in GBM.

### Combining Immunotherapies With Anti-Angiogenic Therapies

There is good preclinical and clinical evidence in a variety of cancer types demonstrating improved survival when combining immunotherapies with agents that target vasculature. For instance, bevacizumab plus interferon-alpha—an immunostimulatory cytokine— is first line therapy in renal cell carcinoma and has been shown to nearly double progression free survival from 5 months to 9-10 months in two phase III clinical trials, as well as objectively increase overall survival (141, 142). In 2018, the pivotal IMpower150 study demonstrated in non-small cell lung cancer that the addition of the PD-L1 inhibitor atezolizumab to bevacizumab and chemotherapy resulted in a 22% reduction of risk of death and a 38% reduction in disease progression compared to bevacizumab and chemotherapy alone (143). There also exists successful phase III data for atezolizumab in combination with bevacizumab for unresectable hepatocellular carcinoma, which demonstrates a 42% reduction in risk of death and 41% reduction in progression when compared to the tyrosine kinase inhibitor sorafenib (144).

There are also a number of clinical trials that have combined immunotherapy and anti-angiogenic agents in GBM (**Table 1**). The recent appreciation of the profound effect that bevacizumab has on tumor biology has resulted in many newer clinical trials stratifying patients that have previously failed bevacizumab into a separate treatment arm than bevacizumab-naïve patients. Some trials evaluate the effectiveness of a certain therapy with and without bevacizumab. Despite the promise of combinatorial therapy, clinical trials for GBM are generally conservative in their approach, as evidenced by bevacizumab being the only anti-angiogenic agent that has so far been trialed with immunotherapy. Bevacizumab does have the theoretical advantage in indirectly promoting an immune response through the reduction in use of corticosteroids (149). But there have been no successful phase III trials yet in immune therapy and anti-VEGF combinatorial treatment for GBM. The two best studied combinations are immune checkpoint inhibitors with bevacizumab and vaccine-based therapies with bevacizumab. However, the development of new regimens will be necessary for future success.

**Table 1:** Clinical trials for glioblastoma with combination immunotherapy and anti-angiogenic therapy.

Year	Immunotherapy	Antiangiogenic Therapy	Phase	Status	Results	ClinicalTrials.gov identifier
* Vaccine Therapies*						
2011	Rindopepimut EGFRvIII Peptide Vaccine with GM-CSF	Bevacizumab	II	Completed	Primary Endpoint: Objective but nonsignificant increase in six-month progression-free survival in experimental group (28%) compared to control group (16%). Statistically significant increase in overall survival (HR 0.53%; CI 0.32-0.88, p=0.001) Discontinuation of steroids after six months was 33% in experimental group and 0% in control group.	NCT01498328 (145)
2013	Heat shock protein peptide complex 96 (HSPPC-96) autologous vaccine	Bevacizumab	II	Active, not recruiting	Primary Endpoint: Statistically significant worse median overall survival in experimental group (7.5 months) compared to control group (10.7 months). HR 2.06%; CI 1.18-3.60; p=0.03 This study was terminated after above interim results. Final publication of data is pending.	NCT01814813 (146)
2013	ERC1671 (gliovac) autologous/allogeniec vaccine with GM-CSF	Bevacizumab	II	Active, not recruiting	Primary endpoint: 12-month overall survival. Interim results demonstrate increased median overall survival of 12 months in experimental group compared to 7.5 months in control group.	NCT01903330 (147)
2014	SL-701 multivalent synthetic TAA vaccine (interleukin-13 receptor alpha-2, ephrinA2, survivin)	Bevacizumab	I/II	Completed	Primary endpoints: objective response rate and 12-month overall survival. Full results not published.	NCT02078648 (40)
2016	Synthetic TAA vaccine (EGFRvIII, iL13Ralpha, ephA2, her2/neu, YKL-40), with poly-ICLC (Toll-like Receptor Agonist) and montanide ISA-51 VG (secondary adjuvant)	Bevacizumab	II	Withdrawn	n/a	NCT02754362
2017	DSP-7888 dosing emulsion (WT1 peptides)	Bevacizumab	III	Recruiting	*Ongoing*	NCT03149003
2019	Autologous DCs pulsed with genetically modified tumor cells or TAA	Bevacizumab	I	Enrolling by invitation	*Ongoing*	NCT03914768
2019	(EO2401) trivalent synthetic TAA vaccine with and without Nivolumab (anti-PD-1)	Bevacizumab	I/II	Recruiting	*Ongoing*	NCT04116658
2020	Autologous DCs pulsed with TAA	Bevacizumab	I/II	Recruiting	*Ongoing*	NCT04277221
* Checkpoint Inhibitors*						
2012	Camrelizumab (anti-PD-1)	Bevacizumab	II	Recruiting	*Ongoing*	NCT04952571
2015	Pembrolizumab (anti-PD-1)	Bevacizumab	II	Completed	Primary endpoint: No significant differences in six-month progression-free survival or median overall survival in experimental group vs control group. In experimental group, worse overall survival correlated with baseline dexamethasone use and increased posttherapy plasma VEGF.	NCT02337491 (148)
2015	Durvalumab (anti-PD-L1)	Bevacizumab	II	Completed	Primary endpoint: 12-month overall survival. Preliminary analysis demonstrated 60% 12-month survival in MGMT-unmethylated GBM compared to 50% in an historical benchmark. Full results are pending.	NCT02336165 (43)
2018	Pembrolizumab (anti-PD-1)	Bevacizumab	II	Active, not recruiting	*Ongoing*	NCT03661723
2018	Nivolumab (anti-PD-1)	Bevacizumab	II	Active, not recruiting	*Ongoing*	NCT03452579
2018	Retifanlimab (anti-PD-1) with and without epacadostat (indoleamine 2,3-dioxygenase inhibitor)	Bevacizumab	II	Recruiting	*Ongoing*	NCT03532295
2019	Nivolumab (anti-PD-1)	Bevacizumab	II	Recruiting	*Ongoing*	NCT03890952

Checkpoint inhibitors that have been tested with bevacizumab in clinical trials are monoclonal antibody inhibitors of PD-1 or PD-L1, including camrelizumab, bembrolizumab, durvalumab, and nivolumab. Two of these trials have completed phase II testing. In one study, pembrolizumab in combination with bevacizumab was ineffective in prolonging overall survival or progression free survival, and no tumor immune biomarkers that were collected (including tumor PD-L1 expression, tumor-infiltrating lymphocyte density, immune activation gene expression signature, and plasma cytokines) predicted outcomes (148). Interestingly, poor survival correlated with increased baseline dexamethasone use and increased posttherapy plasma VEGF, which should be carefully evaluated as potential markers in future combinatorial studies. In a second study, durvalumab in combination with bevacizumab and radiotherapy showed promise among a subgroup of patients with unmethylated MGMT tumors, however full results have yet to be posted (43). An intriguing study that is currently enrolling patients investigates the effect of retifanlimab, a PD-1 inhibitor, with or without epacadostat, an indoleamine 2,3-dioxygenase (IDO) inhibitor, in combination with bevacizumab and radiation in recurrent glioblastoma (150). IDO is an enzyme that catalyzes the rate limiting step of tryptophan (Trp) catabolism, converting Trp to kynurenine (Kyn). It has been demonstrated that Trp depletion and Kyn accumulation leads to immunosuppression by functional inhibition of CD8+ and NK cells, and functional stimulation regulatory T cells (151). The addition of epacadostat may result in a necessary reduction of the immunosuppressive milieu of GBM that enables efficacy of a PD-1 inhibitor with bevacizumab.

There are a number of vaccine therapies that have undergone clinical testing with bevacizumab, including TAA, HSP, and DC vaccines. Rindopepimut, an EGFRvIII-targeted vaccine that consists of a peptide with homology to EGFRvIII that is conjugated to keyhole limpet hemocyanin, is one promising agent that in combination with bevacizumab has completed phase II testing in patients with relapsed EGFRvIII-expressing GBM (145). Although there was a relatively small sample size of 36 patients in the experimental arm, these patients had an improved overall survival compared to control arm (hazard ratio of 0.53), and 33% of patients were able to discontinue steroids compared to 0% in control arm. Another vaccine in combination with bevacizumab that has reached phase II clinical trial is an autologous HSP, HSPPC-96, generated from patient resected tumors (146). However, the study was terminated after interim analysis surprisingly showed worse overall survival in experimental group compared to the control group, and complete results have not yet been published.

ERC1671 (gliovac) is an intriguing immunotherapy that has been tested in combination with bevacizumab, and it consists of autologous inactivated tumor cells lysate from the patient to be treated, inactivated tumor cells and lysate from three other GBM patients, cyclophosphamide to inhibit local immunosuppression, and GM-CSF as an adjuvant to enhance the immune response (147). Interim results show improved median overall survival of 12 months in ERC1671 plus bevacizumab arm, compared to 7.5 months in bevacizumab alone. Additionally, CD4+ T-lymphocyte counts correlated with overall survival. Full results are pending.

SL-701 is a vaccine therapy with adjuvants GM-CSF and imiquimod that has completed phase 2 testing. This vaccine is comprised of synthetic peptides designed to elicit an immune response against interleukin-13 receptor alpha-2, ephrinA2 and survivin (40). Although an initial report suggested a possible survival tail in refractory GBM patients, full data has not been released.

**Table 2** includes the targeted therapies that have been combined with bevacizumab in clinical trials and which involve the selective inhibition of intercellular pathways that are partially implicated in immune signaling. Many of these have unfortunately resulted in disappointing trial results. VB-111’s effect on the immune system is discussed above. One promising agent is abemaciclib, a CDK 4/6 inhibitor, that induces a T-cell inflamed tumor microenvironment, and may also potentiate the effects of bevacizumab through reduction in metabolic invasiveness (154–156).

**Table 2:** Clinical trials for glioblastoma with combination anti-angiogenic therapy and targeted inhibitors implicated in immunity.

Year	Targeted Therapy	Antiangiogenic Therapy	Phase	Status	Results	ClinicalTrials.gov identifier
2008	Tandutinib (ems-like tyrosine kinase 3 antagonist)	Bevacizumab	II	Completed	Primary endpoint: six-month progression-free survival was 23%. Tandutinib with bevacizumab was equally effective but more toxic than bevacizumab monotherapy.	NCT00667394 (152)
2008	Everolimus (mTOR inhibitor)	Bevacizumab	II	Completed	Primary endpoint: median progression free survival was 11.3 months.	NCT00805961 (153)
2008	Temsirolimus (mTOR inhibitor)	Bevacizumab	II	Completed	Primary endpoint: median progression free survival of eight weeks. Trial terminated early because of poor outcomes.	NCT00800917 (153)
2011	Buparlisib (selective PI3K inhibitor)	Bevacizumab	I/II	Completed	Primary endpoint: median progression free survival was 5.3 months. Full results not published.	NCT01349660 (41)
2011	Plerixafor (CXCR4 inhibitor)	Bevacizumab	I	Terminated	n/a	NCT01339039
2015	Ofranergene obadenovec (adenovirus delivering chimeric death receptor	Ofranergene obadenovec/ Bevacizumab	III	Completed	Primary endpoint: median overall survival was 6.8 months in combination arm versus 7.9 months in control arm. Change of treatment regimen, with the lack of VB-111 monotherapy priming, may explain the differences from the favorable phase II results.	NCT02511405 (135)
2018	ABI009 - nanoparticle albumin-bound rapamycin (mTOR inhibitor)	Bevacizumab	II	Active, not recruiting	*Ongoing*	NCT03463265
2019	Abemaciclib (CDK 4/6 inhibitors)	Bevacizumab	I	Recruiting	*Ongoing*	NCT04074785

## Future Directions

Despite the failure so far in individual immunotherapies and anti-angiogenic therapies in GBM, translational experiments have recently shed new light on the crosstalk between the immune and vascular systems in GBM. One study demonstrated that successful treatment of combined anti-VEGFR2 and anti–PD-L1 in breast cancer and pancreatic cancer was correlated with the induction of high endothelial venules (HEV) that resulted in lymphocyte infiltration through activation of lymphotoxin β receptor (LTβR) signaling (157). While combinatorial therapy with anti-VEGFR2 and anti–PD-L1 showed no induction of HEV in a GBM line, LTβR agonists were then trialed which induced HEVs and enhanced function of CD8+ T cells in GBM (157). This study provides good mechanistic evidence of the utility of combinatory therapy, introduces a new therapeutic target, and underscores that possible biomarkers may exist for treatment response, which will likely lead to the further investigations to those factors that may predispose certain therapies to induce formation of HEV with subsequent lymphocyte infiltration. An intriguing report that utilizes the syngeneic GL261 glioma line demonstrates that anti-VEGF therapy in combination with a picornavirus vaccine (that expresses epitope OVA257–264 to enhance antigen specific CD8+ T-cell) resulted in a synergistic treatment response with prolonged overall survival and delayed disease progression compared to the additive individual effects of these therapies (158). Another intriguing report details a screen for immune mutations in response to anti-VEGF treatment in GL261 and KR158B murine glioma lines that revealed a dose-dependent upregulation of immunosuppressive regulatory T-cell genes in response to anti-VEGF (159). Subsequently, Anti-CD25 to eliminate regulatory T-Cells was injected prior to initiation of anti-VEGF therapy and resulted in improved overall survival compared to either therapy alone (159).

Future challenges include the development of new and rational combinations of treatments, utilization of biomarkers for improved allocation of patients to clinical trials with improved therapeutic monitoring, as well as the broadening of agents that target the vasculature in addition to bevacizumab. While it is likely that some strategies to reduce angiogenesis will also decrease immune cell access, optimal synergistic approaches will generate a robust anti-tumor immune response while simultaneously inhibiting vascular growth.

## Conclusion

The GBM immune and vascular landscape is incredibly complex, and it is likely that a ‘magic bullet’ treatment does not exist. Indeed, a more attainable solution is a shift in perspective to view GBM as a chronic disease, in which combinations of therapies are used on multiple fronts to suppress tumor cell invasion, impair delivery of nutrients, and promote an anti-tumor immune response. Combining immunotherapy and antiangiogenic therapy has shown promise in preclinical models and subsets of real-world patients. Further understanding how these agents interact with one another as well as clinical validation of these results will be essential for further progress in GBM treatment.

## Author Contributions

SJ and EC gathered the ideas together and wrote the review. MA provided ideas for the subtopics, edited the manuscript, and provided overall supervision for the manuscript. All authors contributed to the article and approved the submitted version.

## Conflict of Interest

The authors declare that the research was conducted in the absence of any commercial or financial relationships that could be construed as a potential conflict of interest.

## Publisher’s Note

All claims expressed in this article are solely those of the authors and do not necessarily represent those of their affiliated organizations, or those of the publisher, the editors and the reviewers. Any product that may be evaluated in this article, or claim that may be made by its manufacturer, is not guaranteed or endorsed by the publisher.
